# YOLO11-ALi: an improved YOLO11-based model for blueberry target detection from full flowering to fruit expansion in greenhouse environments

**DOI:** 10.3389/fpls.2026.1874611

**Published:** 2026-07-14

**Authors:** Jiarui Zhang, Ye Su, Long Zhang, Haochen Wang, Yongshuai Yang, Jia Lv, Juan Liu, Jie Hu

**Affiliations:** 1 Faculty of Software Technologies, Shanxi Agricultural University, Jinzhong, Shanxi, China; 2 Department of Basic Courses, Shanxi Agricultural University, Jinzhong, Shanxi, China

**Keywords:** attention mechanism, blueberry detection, feature fusion, greenhouse cultivation, YOLO11

## Abstract

The abscission of blueberry floral organs after fruit set is crucial for fruit development and quality, while the closed, high-humidity greenhouse environment often causes withered flower retention, adversely affecting blueberry growth and commercial value. Current blueberry detection technologies focus on maturity classification, health monitoring and automatic picking, leaving a gap in complex target detection during the full flowering to fruit expansion period. To address this, this study proposes YOLO11-ALi, a modified model based on YOLO11, for greenhouse pot-grown northern highbush blueberries. Three key improvements are made to the baseline: replacing the SPPF module with AIFI to reduce small-target detail loss, integrating LSKAttention into C2PSA to form C2PSA_LSKA for enhanced small-target attention, and incorporating iAFF into the neck C3k2 module to improve cross-layer feature fusion efficiency. A dedicated dataset of 4527 images covering full flowering to fruit expansion was constructed, and ablation experiments verified the modules’ roles and synergistic effects. Results show that the integrated model achieves optimal performance: compared with the original YOLO11, precision, recall, mAP@50 and mAP@50:95 are increased by 0.6%, 1.8%, 1.2% and 6.8% respectively, with computational complexity and inference speed meeting real-time field detection requirements. The proposed model provides a reliable technical basis for intelligent monitoring and refined management of greenhouse blueberries.

## 1 Introduction

As a characteristic fruit tree with high nutritional and economic value, blueberry fruit development quality and plant growth status are directly related to the economic benefits of the industry (Blanke, 2024). The fruit set stage represents a critical period in blueberry growth and development, during which the normal abscission of floral organs serves as an essential physiological process to ensure healthy fruit expansion and reduce resource waste (Zhang et al., 2024). The abscission of blueberry floral organs after fruit set is a programmed physiological process synergistically mediated by hormone regulation, cell structure remodeling, nutrient allocation, and environmental signals (Liu et al., 2022) Ethylene and abscisic acid (ABA) can induce the apoptosis of abscission zone cells at the base of floral organs and the degradation of cell walls, while auxins and cytokinins inhibit the formation of abscission zones by delaying organ senescence (Wang et al., 2023). After fruit set, the shift in source-sink relationships makes fruits the priority center for nutrient allocation, which further accelerates the senescence process of floral organs (Yang et al., 2015). Suitable temperature and light conditions can promote physiological metabolism related to abscission, whereas adverse factors such as low temperature and high humidity, as well as cultivar genetic characteristics, may interfere with this process, leading to the retention of withered flowers (Wen et al., 2018). Compared with open-field cultivation, the closed and windless environment of greenhouses lacks natural external forces to assist in abscission, and the high-humidity microenvironment inside the greenhouse tends to exacerbate the retention of withered flowers (Yang et al., 2011). Retained withered flowers not only inhibit fruit expansion and anthocyanin synthesis through physical shading but also serve as an infection vector for pathogens and pests. Meanwhile, they reduce the ventilation and light transmittance of fruit clusters and cause ineffective consumption of plant nutrients, ultimately exerting a significant negative impact on the commercial quality of blueberry fruits and plant growth (She et al., 2024).

In recent years, artificial intelligence technology has developed rapidly, especially in the field of visual detection in agriculture. Deep learning models have achieved remarkable breakthroughs in the detection of small-target fruits such as strawberries and coffee, providing technical references for blueberry fruit recognition. Tituana et al. (2024) designed a strawberry ripeness detection robot based on the YOLOv4 model, achieving a mean Average Precision (mAP) of 80.20% with a processing time of 64.40 ms. Kazama et al. (2024) enhanced the YOLOv8 model by modifying the convolutional block RFCAConv to classify the ripening stages of coffee fruits, increasing the mAP by 1.90% without affecting computational resources. Beyond fruit ripeness classification, machine learning and computer vision techniques have also been widely explored for target detection in diverse agricultural crops and complex scenarios. Jing et al. (2024) applied the improved Sunflower-YOLO model to detect sunflower heads in UAV remote sensing images, distinguishing open, semi-open, and bud growth states, and further established a sunflower head density map to support growth monitoring. Zhao et al. (2025b) integrated super-resolution reconstruction with semi-supervised YOLOv10 to improve the detection accuracy of small targets in turbid underwater environments. Li et al. (2025) combined image restoration optimization with an enhanced YOLO framework to realize reliable UAV-based monitoring for succulent plants. You et al. (2025) optimized the YOLOv11 architecture using customized feature extraction modules to achieve robust rose detection under complex greenhouse conditions. Lin et al. (2026) developed a lightweight YOLO detection framework that meets the deployment requirements of edge devices for intelligent selective harvesting in agricultural production.

In the field of blueberry visual detection, relevant technical research has also formed a relatively mature system, with continuous emergence of solutions for scenarios such as blueberry health monitoring, quality analysis, and positioning recognition. Song et al. (2025) proposed an enhanced LBSR-YOLO algorithm integrating BSRN and YOLOv10n. To address the problems of high cost and low efficiency in blueberry health monitoring under intensive cultivation, the algorithm improves the small-target detection capability by modifying convolutional modules and optimizing the network neck structure, and is deployed on edge computing nodes of wireless sensor networks (WSNs), realizing low-cost, low-energy-consumption, and efficient blueberry health monitoring. Shanthini et al. (2025) proposed a hybrid CNN-Transformer architecture model (NorBlueNet) based on hyperspectral imaging to meet the demand for non-destructive detection of soluble solids content (SSC) in Norwegian wild blueberries. By combining the advantages of CNNs in local feature extraction and Transformers in capturing global relationships, the model achieves high-precision prediction of blueberry SSC. Yang et al. (2022) proposed a blueberry recognition model based on improved YOLOv5 to solve the problem that dense adhesion and severe occlusion during blueberry growth hinder automatic picking. Through constructing a blueberry dataset, designing the NCBAM attention module to improve the feature extraction capability of the backbone network, adding a small-target detection layer to enhance multi-scale recognition, and introducing the C3Ghost module to reduce parameters, the model achieves an mAP of 83.2% on a self-built dataset, effectively improving blueberry positioning accuracy to facilitate automatic picking. Xu et al. (2025) proposed the GLL-YOLO method based on YOLOv8 to address the issues of traditional blueberry maturity detection relying on manual work which is inefficient and highly subjective and existing deep learning methods requiring large models with complex computations making real-time deployment on resource-constrained edge devices difficult. Using GhostNetV2 as the backbone network, replacing the original C2f module with the LIMC module, and designing a lightweight shared convolutional detection head (LSCD), the method can accurately detect blueberries at three maturity stages. While improving detection accuracy, it reduces model parameters, floating-point operations (FLOPs), and model size by 50%, 39%, and 46.7%, respectively, providing support for intelligent blueberry picking. Zhao et al. (2025a) further combined Mamba-based super-resolution reconstruction with semantic segmentation to achieve accurate blueberry maturity assessment, promoting the development of intelligent monitoring in precision agriculture.

To this end, this paper proposes YOLO11-ALi, a detection model for blueberries spanning the full flowering to fruit expansion stages, which is based on YOLO11. Specifically, the SPPF module is replaced with the AIFI module, which fuses multi-branch features via adaptive weighting to mitigate the loss of small-target details during pooling. The C2PSA module is improved by integrating the LSKAttention module, which combines the global receptive field of large-kernel convolution with the local detail-capturing capability of small-kernel convolution, thereby enhancing the attention focusing precision on small targets in complex backgrounds. Additionally, the iAFF module is incorporated into the neck C3k2 module; leveraging its iterative attention-driven cross-layer feature fusion mechanism, it overcomes the constraint of single-level feature processing, elevates the feature fusion efficiency of the C3k2 module, and further strengthens the model’s performance in identifying and locating small targets. For data support, a dataset of northern highbush blueberries in greenhouses during the full flowering and fruit expansion stages is constructed. This study can specifically address the detection challenges of northern highbush blueberries in greenhouses from the full flowering to fruit expansion stages, providing a practical technical reference for small-target recognition within this specific cultivation environment and growth period.

## 2 Materials and methods

### 2.1 Dataset collection

**Figure 1:**
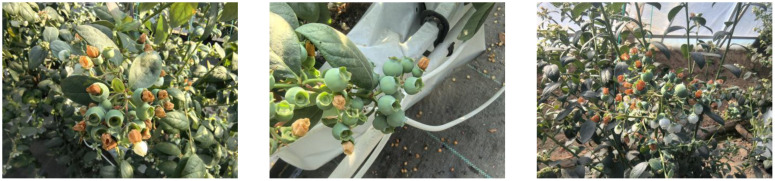
Examples of blueberry images.

The experimental data were collected from Shanxi Province. The research material was Vaccinium corymbosum, northern highbush blueberry, cultivated in substrate filled pots under greenhouse conditions. Each pot had a diameter of 50 cm, with a plant spacing of 50 cm and a row spacing of 150 cm. Blueberry plants were randomly selected as research subjects. During the period from full bloom to fruit expansion, multi-distance image sampling was conducted every 3 days at three fixed distances: 30 cm, 50 cm, and 70 cm. Images were captured using a mobile phone with a resolution of 4000×3000 pixels, as shown in **Figure 1**, effectively preserving scene details such as fruit shape and peel texture, which meets the basic image quality requirements of the study.

### 2.2 Dataset classification

After screening out blurred images, a total of 1509 original images were obtained. These original data were first divided into training, validation, and test sets at an 8:1:1 ratio to avoid data leakage. Data augmentation techniques were then applied only to the training set, including random flipping, rotation, and brightness adjustment. This process expanded the training set to three times its original size, and the final total number of images in the whole dataset reached 4527.

Three classes were defined for annotation, denoted as a, b, and c, with clear biological and morphological significance. Class a corresponds to post-anthesis fruits with faded and persistent yellow floral tissues. Class b corresponds to early-stage expanding fruits with completely abscised floral organs and a smooth fruit apex. Class c corresponds to fully bloomed flowers before fruit setting. The primary morphological difference between class a and class b lies in the presence or absence of residual floral structures at the fruit apex, which serves as a consistent and reliable criterion for annotation.

**Figure 2:**
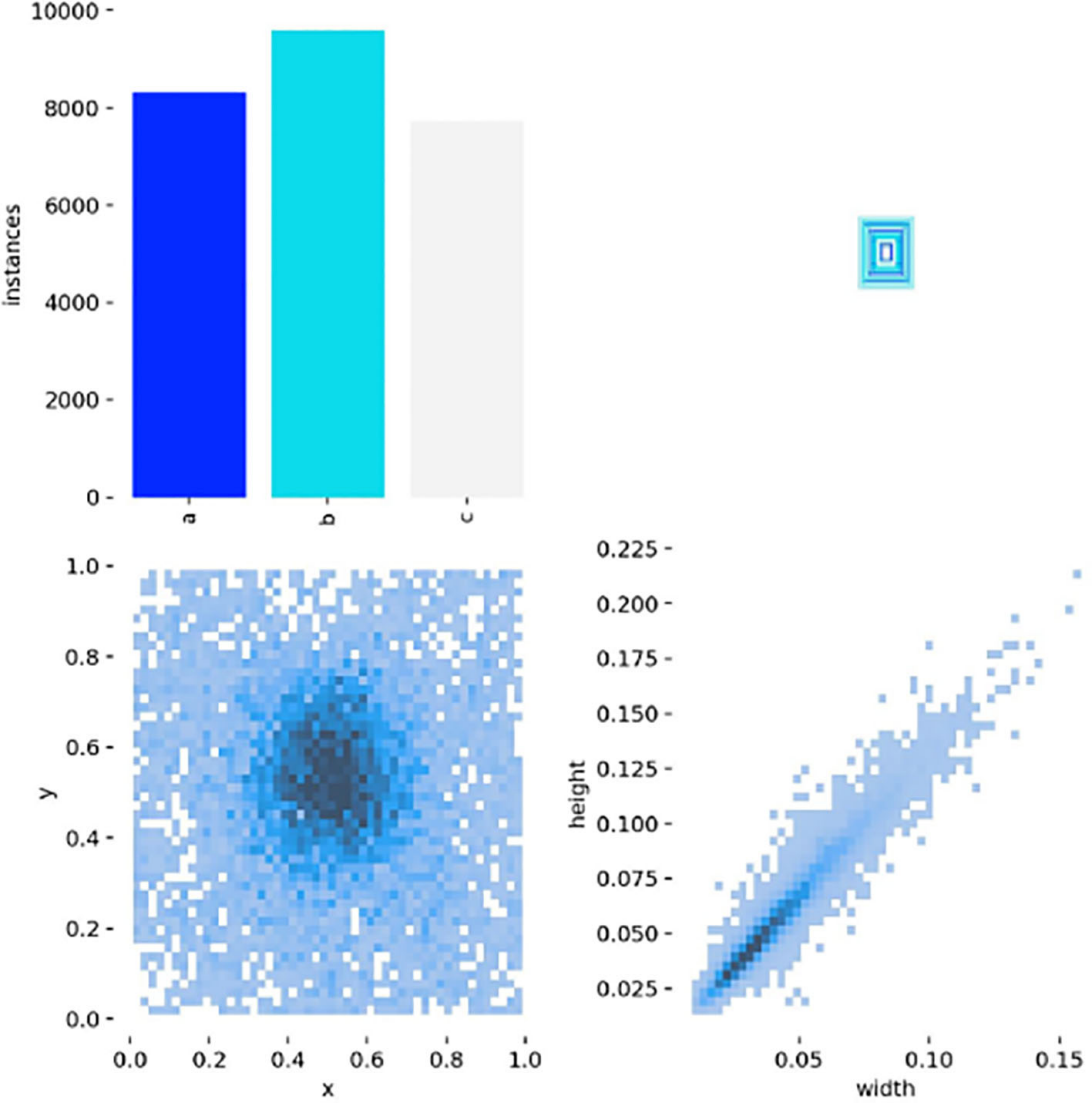
Blueberry dataset statistics.

A total of 8256 instances were labeled for class a, 9683 for class b, and 7662 for class c. The class distribution and target size characteristics of the dataset are illustrated in **Figure 2**. Standardized annotation rules were applied. Bounding boxes tightly enclosed each target, and partially occluded objects were labeled as long as key morphological characteristics remained distinguishable. All annotations were verified through cross-checking between annotators and further reviewed by professional agricultural technicians to ensure accuracy and consistency.

### 2.3 Improvement methods

Compared to other YOLO series models, YOLO11 optimizes the backbone and neck network architectures, significantly reducing parameters while enhancing feature extraction capabilities, thus achieving higher detection accuracy and faster inference speed (Khanam and Hussain, 2024). Its multi-task support and cross-environment deployment adaptability further expand application scenarios (He et al., 2024). Additionally, advanced technical optimizations such as mixed-precision training and adaptive anchor box mechanisms improve training efficiency and target adaptability, making YOLO11 particularly outstanding in resource-constrained and real-time-required scenarios (Zhao et al., 2025). It thus serves as an ideal baseline model balancing performance and flexibility.

**Figure 3:**
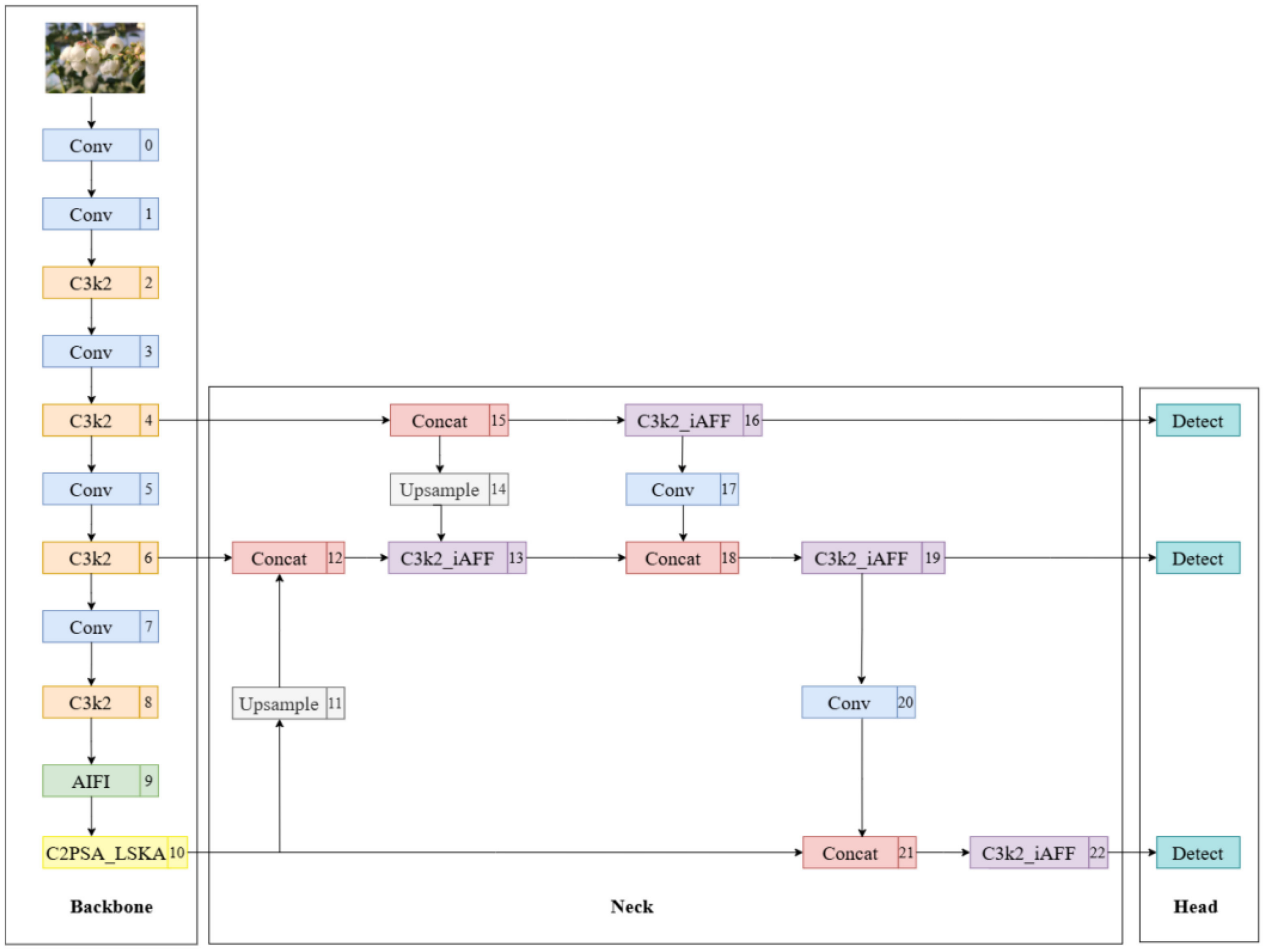
YOLO11-ALi Network Architecture.

In this study, we employ existing mature modules and the standard YOLO11n framework to perform task-oriented integration and optimization for blueberry small-object detection, developing the improved YOLO11-ALi model as illustrated in the **Figure 3**.

The original SPPF module in the backbone is replaced by the Adaptive Information Fusion Inception (AIFI) module at the 9th layer of the YOLO11 backbone with a channel dimension of 1024 and 8 attention heads. This module adopts a multi-branch structure: while retaining multi-scale feature extraction, it fuses feature information from different branches using adaptive weights. This design reduces the loss of small-target details during pooling and strengthens small-target feature preservation. To enhance the C2PSA module, the Large-Small Kernel Attention (LSKAttention) module is incorporated to construct the C2PSA_LSKA module, which is deployed immediately after AIFI at the 10th layer of the backbone with a channel setting of 1024. This optimized module integrates the global receptive field of large-kernel convolution and the local detail capture ability of small-kernel convolution. By dynamically assigning feature contributions through attention weights, it improves the accuracy of attention focusing on small-target regions in complex backgrounds and enhances small-target feature signals. Furthermore, the Inter-Layer Feature Fusion (iAFF) module is integrated into the C3k2 modules in the network neck to form C3k2_iAFF, which is applied at layers 13, 16, 19, and 22 of the YOLO11 head with channel parameters of 512, 256, 512, and 1024 respectively. Using a cross-layer interactive feature fusion mechanism, it overcomes the limitation of single-layer feature processing in the original C3k2 module, facilitates sufficient fusion of deep semantic features and shallow detailed features from the backbone, improves the feature fusion efficiency of the neck C3k2 module, and further boosts the model’s feature recognition and localization performance for small targets.

#### 2.3.1 AIFI

**Figure 4:**
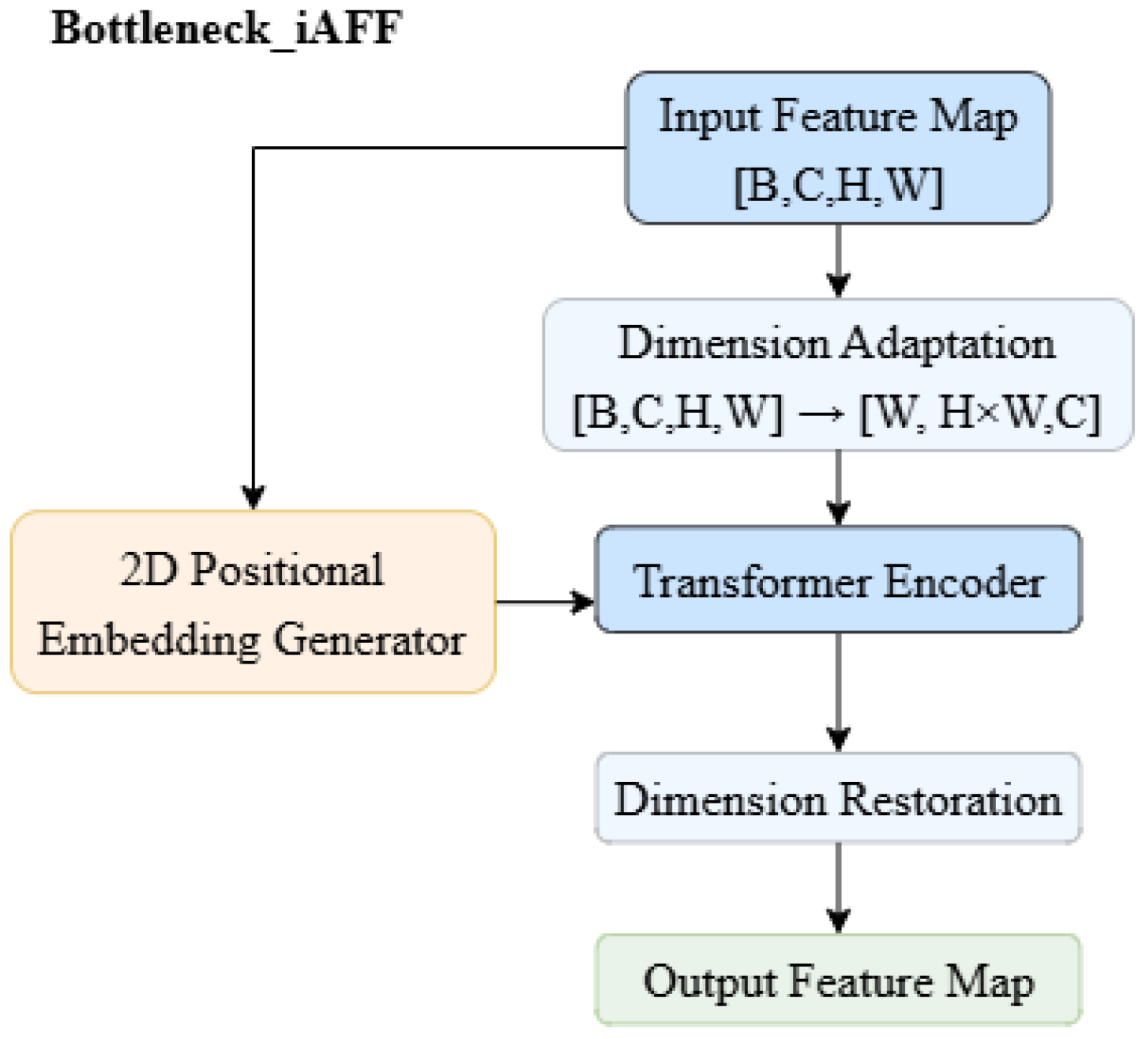
AIFI Network Architecture.

In YOLO11, the SPPF module performs multi-scale spatial pyramid pooling on the feature maps output by the backbone network. By integrating features from receptive fields of varying sizes, it enhances the model’s capability to extract features of targets with different dimensions. As a feature enhancement module, the C2PSA module introduces a parallel spatial attention mechanism during feature propagation, which strengthens the feature weights of key target regions while suppressing redundant background information. The multi-scale features generated by SPPF serve as the foundation for C2PSA, and C2PSA further filters out effective features, thereby jointly improving the model’s feature representation performance. However, in environments with complex background textures, the model exhibits suboptimal detection performance for small targets. Due to the inherently weak feature information of small targets, detailed features are prone to loss after SPPF pooling. Moreover, the limited positioning accuracy of C2PSA on small target regions leads to high missed detection rates and low detection precision for small targets. To address these drawbacks of SPPF, it is replaced by the AIFI module, whose structure is shown in the **Figure 4**.

The core architecture of the AIFI module consists of basic components and customized extended components (Zhao et al., 2024). The basic components reuse the core modules of the Transformer, including a multi-head attention layer that captures global feature dependencies, a feed-forward network that performs nonlinear transformation on the output features of the attention layer, and layer normalization and dropout layers that support pre-normalization and post-normalization modes to alleviate gradient vanishing and reduce overfitting, thus ensuring the feature interaction capability of the Transformer. The customized extended components are newly added to adapt to the input of feature maps in visual tasks: one is a 2D positional embedding generator based on sine-cosine functions, which injects spatial positional information into the Transformer attention layer to address its position invariance issue; the other is a dimension adaptation interface that realizes the conversion between 4D feature maps and 3D tensors required by the Transformer input, ensuring the module can be seamlessly integrated into the feature processing pipeline of the YOLO11 model.

Through the combined design of multi-head attention and 2D positional embedding, the AIFI module not only leverages the Transformer attention mechanism to capture the global dependencies of feature maps, overcoming the limitation of traditional SPPF modules that rely solely on pooling for local feature extraction, but also preserves pixel-level local spatial information via positional embedding. This enables the synergistic modeling of global semantic correlation and local detail localization, which can effectively reduce the loss of detailed features of small targets during feature processing.

#### 2.3.2 C2PSA_LSKA

**Figure 5:**
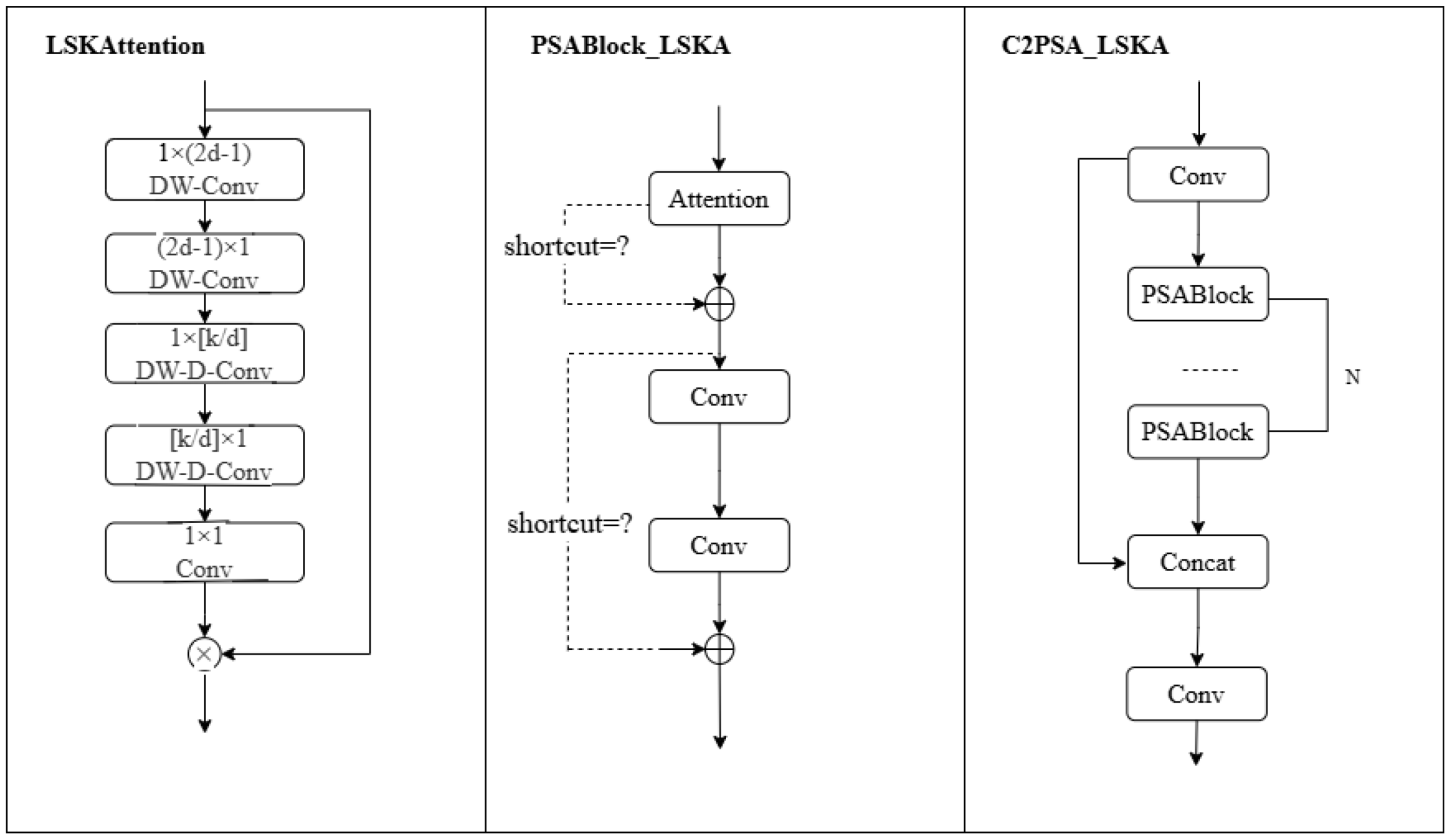
C2PSA_LSKA Network Architecture.

Considering the synergistic mechanism between the SPPF and C2PSA modules, the original C2PSA module exhibits poor compatibility with the improved AIFI module during feature interaction. Meanwhile, the C2PSA module itself suffers from limited attention focusing accuracy on small target regions in complex backgrounds. To address these issues, this study further introduces the LSKAttention module to optimize C2PSA. By integrating the global receptive field of large-kernel convolution and the local detail capture capability of small-kernel convolution, the module’s performance in screening and enhancing small target features is improved. The optimized module is named C2PSA_LSKA, whose structure is shown in the **Figure 5**.

The C2PSA_LSKA module optimizes the small target feature capture capability by integrating the LSKA mechanism. It splits the input features into a direct branch and an enhancement branch along the channel dimension via convolution. The enhancement branch is processed by multiple serially connected PSABlocks, where each PSABlock consists of an LSKAttention layer and a feed-forward network, with residual connections supported to strengthen feature propagation. Finally, the direct branch and the enhanced branch are concatenated to achieve the synergy of basic feature preservation and target feature enhancement.

The LSKAttention module is a lightweight attention module designed for visual feature enhancement (Lau et al., 2024). It takes the feature channel dimension and a hyperparameter (k) as initialization parameters, and configures differentiated horizontal and vertical grouped convolution layers according to different k values. Separate 1D convolutions are adopted to capture spatial dependencies in the horizontal and vertical dimensions of feature maps, respectively. Meanwhile, combined with the combined design of different dilation rates and convolution kernel sizes, it adapts to the requirements of spatial receptive fields at various scales. In the forward propagation process, the input feature map first undergoes initial horizontal and vertical convolutions to complete basic spatial feature extraction, then uses high-dilation-rate spatial convolutions to further expand the receptive field for capturing long-range spatial correlations, and finally employs a 1×1 convolution to realize dimension integration and normalization of attention weights. Ultimately, the generated spatial attention weights are multiplied element-wise with the original input feature map, thereby enhancing key feature regions and suppressing redundant background information.

#### 2.3.3 C3k2_iAFF

**Figure 6:**
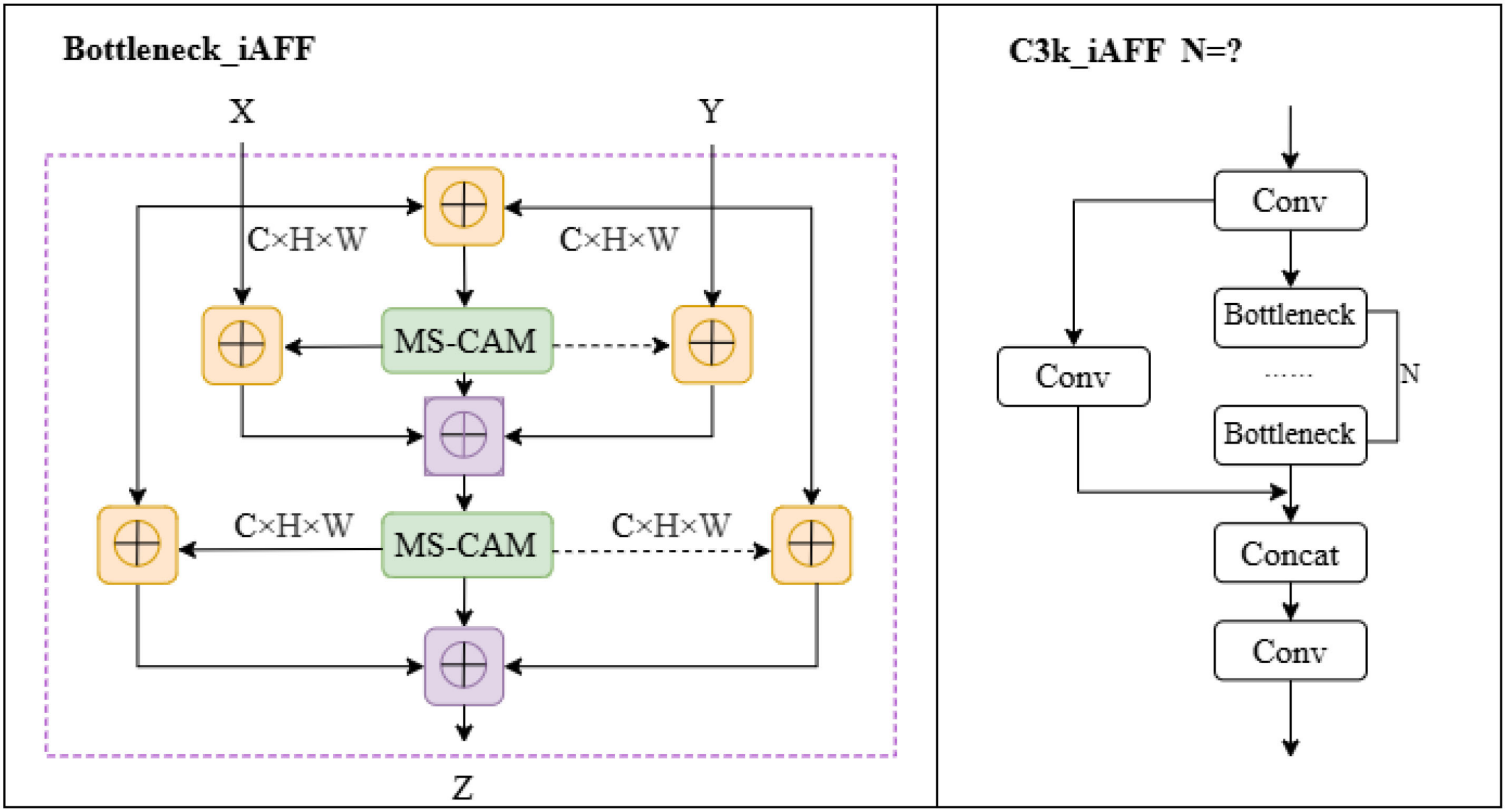
C3k2_iAFF Network Architecture.

The basic feature integration capability of the original neck C3k2 module fails to fully exploit the richer and more refined target feature information extracted by the improved backbone network, resulting in poor compatibility. The module lacks a refined optimization mechanism for the feature integration process, leading to rough initial feature integration performance for small targets in the blueberry classification task and making it difficult to effectively enhance key features such as corolla contours, calyx residues, and peel textures. Meanwhile, the module does not have the ability to dynamically adjust feature weights, which cannot suppress redundant information from complex backgrounds, such as leaf occlusion and light/shadow interference. Consequently, small target features are prone to dilution during the fusion process, thereby reducing the model’s target discrimination and localization accuracy. To address these issues, this study integrates the iAFF module with the C3k2 module and proposes the C3k2_iAFF module, whose structure is shown in the **Figure 6**.

The C3k2_iAFF module optimizes the feature integration performance for small targets by incorporating the iAFF mechanism. Based on the C2f architecture, the module first splits the input features into two branches along the channel dimension via 1×1 convolution. The core branch is processed by multiple serially connected C3k or Bottleneck_iAFF modules, where each Bottleneck_iAFF consists of a two-layer convolution and an iAFF feature fusion layer. It iteratively optimizes feature weight allocation through local-global attention and supports residual connections to strengthen feature propagation. Finally, features from all branches are concatenated and fused via convolution, achieving the synergy of key small target feature enhancement and background redundancy suppression.

The iAFF module is an iterative attention mechanism module designed for visual feature fusion, aiming to optimize feature integration effects through multi-round attention interaction (Dai et al., 2021). This module takes the number of feature channels and channel compression coefficient as core parameters, and constructs two groups of symmetric local-global attention branches. The local attention branch captures the local spatial correlations of features via two-stage 1×1 convolutions, while the global attention branch extracts global channel-level feature information by combining adaptive average pooling with 1×1 convolutions. During the forward propagation process, the module first sums the input features and residual features to obtain initial fusion features, generates attention weights through the first round of attention computation, and achieves adaptive fusion of input features and residual features via weight assignment. Subsequently, it performs the second round of attention computation on the fused features, generates secondary optimization weights, and completes the iterative fusion of features. Finally, it outputs the fused features optimized by two rounds of attention. Through iterative attention weight assignment, this module can dynamically enhance key features and suppress redundant information, effectively improving the retention and enhancement effects of small-target features during the fusion process.

### 2.4 Experimental environment and training parameters

#### 2.4.1 Experimental environment

To ensure efficient and stable model training and performance testing, the experimental environment constructed in this study is configured as follows: In terms of hardware, an NVIDIA GeForce RTX 4080 graphics card is used to provide GPU computing power, paired with 64G memory (RAM) to meet the requirements of large-scale data processing and model parameter loading. The operating system adopted is Windows 10, and the software framework is built based on PyTorch 2.2.0 with cu118 version, which is compatible with the CUDA 11.8 computing platform. This ensures the effective activation of GPU acceleration, providing hardware and software foundations for the training iteration and metric calculation of the YOLO target detection model.

#### 2.4.2 Training parameters

Considering the computing power of the experimental environment and the scale of the dataset, the training parameters are set as follows: The batch size is set to 16, which is adapted to the memory capacity of the RTX 4080 graphics card, ensuring training efficiency while avoiding memory overflow. The number of data loading workers is set to 6, making full use of the 64G memory advantage to reduce data reading waiting time and improve training iteration speed. The input image size is uniformly set to 640 pixels. The initial learning rate is set to 0.01, and a cosine annealing learning rate scheduling strategy is adopted with a cycle length equal to the total training epochs (250) and a minimum learning rate of 0.0001 to gradually reduce the learning rate in the later stage of training to promote model convergence. The AdamW optimizer is selected, with a weight decay coefficient of 0.0005 to suppress model overfitting. Automatic mixed precision training is enabled to balance training speed and model stability. The total number of training epochs is set to 250, with model weights saved once per epoch, and performance changes monitored on the validation set simultaneously.

### 2.5 Evaluation metrics

After training the target detection model based on the above dataset, six core metrics are employed to evaluate its performance: Precision (P) reflects the accuracy of the model’s predictions to avoid misjudgment of non-targets; Recall (R) denotes the model’s ability to cover target recognition and reduce missed detections; mAP@50 reflects the model’s overall recognition performance for various blueberry target categories when IOU = 0.5; mAP@50:95 indicates the model’s stability under varying detection accuracy requirements; GFLOPs represents the model’s computational overhead, with a lower value indicating better adaptability to edge computing devices; and FPS reflects the model’s real-time detection capability, with a higher value better meeting the dynamic detection needs of mobile acquisition devices and thus improving detection efficiency.

## 3 Experimental results and analysis

### 3.1 Experimental results

To verify the practical performance of YOLO11-ALi in the multi-category detection task of northern highbush blueberries, model testing was conducted based on the aforementioned dataset, experimental environment and training parameters, with the results presented in **Table 1**.

**Table 1:** Experimental results of YOLO11-ALi.

Classes	P(%)	R(%)	mAP@50(%)	mAP@50:95(%)	GFLOPs	FPS
all	91.1	84.5	93.2	77.7	6.7	61.7
a	89.8	82.3	92.0	74.9		
b	91.5	86.1	93.6	79.8		
c	92.0	85.2	94.1	78.5		

As can be seen from the data in the ‘all’ row, the model achieved a precision, recall and F1-score of over 84.5% in the multi-category detection task of northern highbush blueberries, with the mAP@50 reaching 93.2% and the mAP@50:95 attaining 77.7%. These results demonstrate that YOLO11-ALi can effectively balance the requirements of accurate recognition and comprehensive coverage, exhibiting stable detection performance for potted northern highbush blueberries in greenhouse environments and satisfying the basic accuracy demands of greenhouse target detection tasks. In terms of the detailed results across the three categories, the model performance was relatively balanced yet still presented certain discrepancies. Such differences stemmed from the unbalanced difficulty levels of sample recognition: the model can learn feature representations more sufficiently for categories with moderate recognition difficulty, whereas categories with higher complexity are prone to misjudgment in scenarios involving occlusion or backlighting due to the challenges in feature learning. Moreover, the model had a computational complexity of 6.7 GFLOPs, indicating a relatively low level of computational overhead that does not require high-end hardware support. This characteristic enables its subsequent integration into portable orchard detection devices. With an FPS of 61.7, and benefiting from the optimization of the Windows 10 operating system and PyTorch 2.2.0 framework, YOLO11-ALi is capable of real-time response. This avoids efficiency degradation in on-site orchard operations caused by detection latency, thus meeting the speed requirements of practical application scenarios.

**Figure 7:**
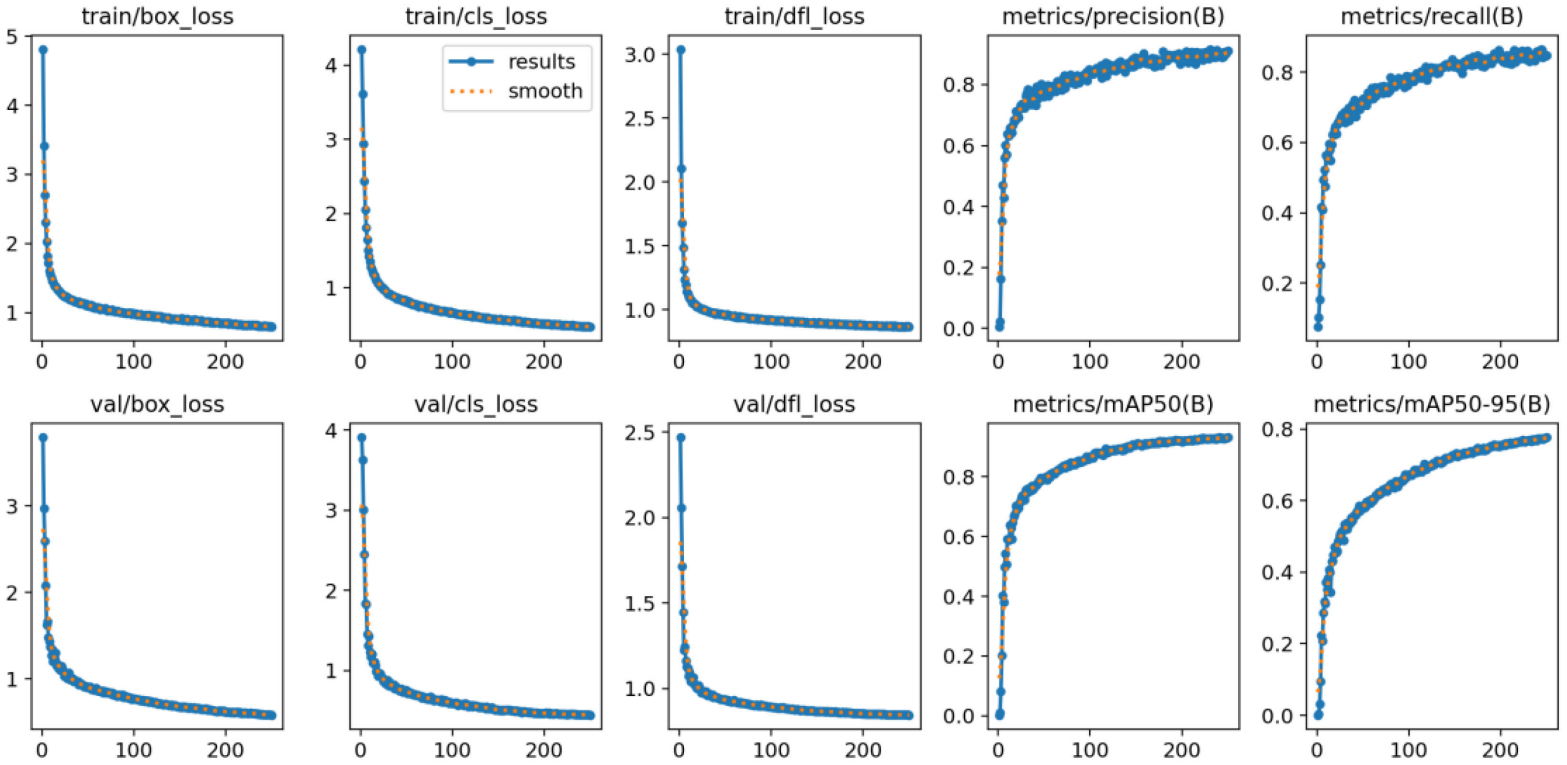
Figure of Model Training and Evaluation Results.

To comprehensively assess the stability of the model training process and the evolutionary pattern of its performance, and to further validate the reliability of the experimental results, we plotted curves illustrating the training loss and variations in key performance metrics, as shown in **Figure 7**, covering the loss changes and performance indicator iteration trends during the training phase.

The first three training loss curves in the first row all show a trend of rapid decline followed by stable convergence, reflecting high learning efficiency, fast parameter optimization, and stable convergence in the early stage of model training. The first three validation loss curves in the second row are highly consistent with the training loss trend, and their values do not increase significantly, indicating that the model has no overfitting problem, possesses good generalization ability, and the training process is reliable. Meanwhile, the P, R, mAP@50, and mAP@50:95 curves on the right also rise rapidly and then stabilize, demonstrating that the model’s detection performance has stabilized at a high level, and the overall training effect is healthy and up to standard.

### 3.2 Ablation experiments

This study conducted ablation experiments to thoroughly explore the individual roles and synergistic effects of three modules, AIFI, C2PSA_LSKA, and C3k2_iAFF, in blueberry detection tasks. These three modules are denoted as Module A, Module B, and Module C respectively, with the results presented in **Table 2**. When Module A was integrated independently, the recall and mAP@50:95 of the model were improved, indicating that it has a significant effect on suppressing missed detections of occluded targets caused by the leaves of blueberry plants in greenhouse pot culture scenarios. When Module B was integrated alone, although the mAP@50 decreased slightly, the mAP@50:95 was increased by 2.5%, which demonstrates that this module is adept at extracting fine-grained features of fruits in different growth stages of northern highbush blueberries. When Module C was applied independently, only the mAP@50:95 was substantially improved, while the gains in other metrics were negligible, reflecting its targeted role in optimizing the feature representation of medium-to-high confidence blueberry targets. When the three modules were used in pairwise combinations, the mAP@50:95 was increased by nearly 5%, highlighting the significant synergistic and complementary effects among the modules, which can integrate target positioning, feature enhancement and anti-interference capabilities to adapt to the complex background of greenhouse pot culture environments. The optimal performance of the model was achieved when all three modules were integrated simultaneously: compared with the original model, the P, R, mAP@50 and mAP@50:95 were improved by 0.6%, 1.8%, 1.2% and 6.8% respectively, which verifies the superiority of this combination in high-precision and full-cycle detection of northern highbush blueberry fruits in greenhouse pot culture. Although the computational complexity increased by 0.4 GFLOPs and the inference speed decreased to 61.7 FPS, the model still meets the practical requirements for real-time inspection using field-portable devices.

**Table 2:** Ablation experiment results.

Model	Module			P(%)	R(%)	mAP@50(%)	mAP@50:95(%)	GFLOPs	FPS
	A	B	C						
1				90.5	82.7	92.0	70.9	6.3	93.3
2	✓			89.1	85.0	92.0	71.2	6.6	78.2
3		✓		88.4	84.9	91.8	73.4	6.3	94.7
4			✓	91.4	82.9	92.5	73.9	6.4	84.8
5	✓	✓		89.5	85.4	93.0	76.0	6.6	79.1
6	✓		✓	90.3	84.6	92.4	75.7	6.7	72.0
7		✓	✓	88.3	87.5	93.1	76.0	6.4	85.8
8	✓	✓	✓	91.1	84.5	93.2	77.7	6.7	61.7

### 3.3 Control experiments on the independent and synergistic effects of C3k2 modules

A total of four C3k2 modules are configured in the neck of the model, which are deployed at layers 13, 16, 19 and 22 of the network, respectively. Their specific distribution is shown in **Figure 3**. Layer 16 is designated as the small-target detection layer, layers 13 and 19 serve as the medium-target detection layers, and layer 22 functions as the large-target detection layer. Among these layers, layer 13 acts as a feature transition layer, while layers 16, 19 and 22 are directly connected to the detection heads. To investigate the impact of modifying C3k2 modules at different positions on blueberry detection performance, this study designed a series of control experiments. Specifically, the experiments were carried out in three steps: first, verifying the performance effects of independently modifying each of the four layers; second, analyzing the synergistic optimization effects between layer 13 and the other three layers; third, conducting experiments by removing the layer 13 and only modifying the modules of the remaining three layers; finally, conducting performance tests on the joint modification of all four layers. The experimental results are presented in **Table 3**.

**Table 3:** Control experimental results of C3k2 models.

Module				P(%)	R(%)	mAP@50(%)	mAP@50:95(%)
13	16	19	22				
				90.5	82.7	92.0	70.9
✓				90.3	83.3	92.3	76.8
	✓			89.5	82.8	91.8	76.3
		✓		88.7	83.7	92.0	76.6
			✓	90.8	82.8	92.4	76.9
✓	✓			88.9	83.6	92.0	76.4
✓		✓		89.4	82.4	92.1	76.2
✓			✓	90.3	83.3	92.3	76.8
	✓	✓	✓	89.0	82.9	91.2	76.5
✓	✓	✓	✓	91.1	84.5	93.2	77.7

The experimental results demonstrate that the performance improvement of the model in blueberry detection tasks is relatively limited when modifying the C3k2 module at a single position independently or performing collaborative modification on the module at layer 13 and that at only one of the other three layers. Notably, modifying only the 16, 19, and 22 layers without the layer 13 C3k2 module yields worse performance than the pairwise combination of layer 13 and the other layers, verifying the indispensable feature transition role of layer 13. Only when the C3k2 modules at all four positions are jointly optimized in a synergistic manner can the model achieve the optimal improvement in detection accuracy and robustness. Therefore, the synergistic modification scheme of the four-layer C3k2 modules is determined as the optimal solution in this study.

### 3.4 Comparative experiments with mainstream YOLO models

To verify the performance advantages of the YOLO11-ALi model and eliminate interference from intergenerational differences in model architectures on the results, a horizontal comparative experiment was conducted between YOLO11-ALi and similar models including YOLOv5, YOLOv6, YOLOv8, YOLOv9, YOLOv10,and YOLO11. All models were trained and tested under the same experimental environment, identical training parameters, and the same dataset to ensure fair and consistent experimental conditions. The experimental results are presented in **Table 4**.

**Table 4:** Performance and efficiency comparison of YOLO models.

Model	P(%)	R(%)	mAP@50(%)	mAP@50:95(%)	GFLOPs	FPS
YOLOv5	86.3	80.5	89.1	67.0	5.8	108.8
YOLOv6	89.8	83.0	90.8	69.8	11.5	106.5
YOLOv8	90.5	82.8	91.6	71.2	6.8	100.7
YOLOv9	89.6	83.1	90.9	70.7	6.4	63.0
YOLOv10	88.8	82.5	91.3	70.8	8.2	83.0
YOLO11	90.5	82.7	92.0	70.9	6.3	93.3
YOLO11-ALi	91.1	84.5	93.2	77.7	6.7	61.7

As can be seen from the data in the table, among the baseline models ranging from YOLOv5 to YOLO11, YOLO11 exhibits a relatively comprehensive performance. Its precision is on par with that of YOLOv8, both reaching 90.5%, and its mAP@50 ranks first among the baseline models at 92.0%. Meanwhile, YOLO11 maintains a low computational complexity of merely 6.3 GFLOPs, achieving a favorable balance between detection accuracy and computational efficiency. The YOLO11-ALi model, which is further improved based on YOLO11, clear improvements across all detection metrics, showing enhanced performance over the series of baseline models. In terms of efficiency, YOLO11-ALi exhibits a slightly higher computational complexity and a moderate reduction in inference speed compared to YOLO11. Despite this trade-off, it still satisfies the practical requirements for real-time detection of potted northern highbush blueberries in greenhouse environments.

To ensure statistical robustness, all comparative models were trained and tested three independent times under identical experimental settings, with results reported as mean ± standard deviation, the results are presented in **Table 5**. The proposed YOLO11-ALi exhibits better performance across all evaluation metrics than the baseline detectors. In addition, the small standard deviations across repeated runs indicate that the performance differences are stable and less affected by random seed initialization, which supports the reliability of the experimental conclusions.

**Table 5:** Statistical performance comparison of YOLO models.

Model	P(%)	R(%)	mAP@50(%)	mAP@50:95(%)
YOLOv5	87.04 ± 1.06	80.68 ± 0.99	91.24 ± 0.40	66.84 ± 0.51
YOLOv6	89.85 ± 0.31	83.06 ± 0.29	90.61 ± 0.32	69.76 ± 0.26
YOLOv8	90.67 ± 0.88	83.05 ± 0.24	91.80 ± 0.39	71.65 ± 0.25
YOLOv9	90.07 ± 1.21	82.59 ± 0.68	91.01 ± 0.27	70.46 ± 0.69
YOLOv10	89.23 ± 0.84	81.20 ± 0.66	91.49 ± 0.27	71.09 ± 0.34
YOLO11	90.37 ± 0.42	82.26 ± 0.63	92.01 ± 0.55	70.65 ± 0.44
YOLO11-ALi	91.15 ± 0.27	84.16 ± 0.59	92.18 ± 0.10	78.31 ± 0.22

### 3.5 Comparison experiments of different lightweight feature enhancement modules

To address the concern regarding the computational complexity and engineering deployment value of the AIFI module, and to verify its rationality compared with other lightweight structures, this study further conducted comparative experiments on several representative feature enhancement modules, including SPPFCSPC, SPPELAN, and FocalModulation. All modules were integrated into the baseline network under consistent experimental settings, with no other structural changes introduced, to ensure a fair comparison of detection performance, computational complexity, and inference efficiency. The experimental results are presented in **Table 6**.

**Table 6:** Comparison results of different lightweight feature enhancement modules.

Model	P(%)	R(%)	mAP@50(%)	mAP@50:95(%)	GFLOPs	FPS
SPPFCSPC	92.8	81.7	91.2	70.6	7.6	86.0
SPPELAN	87.9	82.8	90.6	69.4	6.2	92.9
FocalModulation	85.3	81.1	88.4	67.4	6.4	81.4
AIFI	89.1	85	92.0	71.2	6.6	78.2

The AIFI module achieves the optimal comprehensive detection performance among all compared modules, with the highest mAP@50 of 92.0% and mAP@50:95 of 71.2%, effectively alleviating the small-target detail loss caused by conventional pooling structures. Although the computational complexity of AIFI is slightly higher than that of SPPFCSPC and SPPELAN, its GFLOPs remain at a low level of 6.6, and the FPS is maintained at 78.2, which still satisfies the real-time requirements for practical deployment. In contrast, other lightweight modules fail to achieve a favorable balance between accuracy and complexity, with either insufficient detection precision or limited improvement in small-target characterization. Therefore, the introduction of the AIFI module is reasonable and effective, as it significantly enhances feature representation capability with acceptable additional computational overhead, making it suitable for engineering applications in blueberry detection scenarios.

### 3.6 Visualization

#### 3.6.1 Analysis of detection results

**Figure 8:**
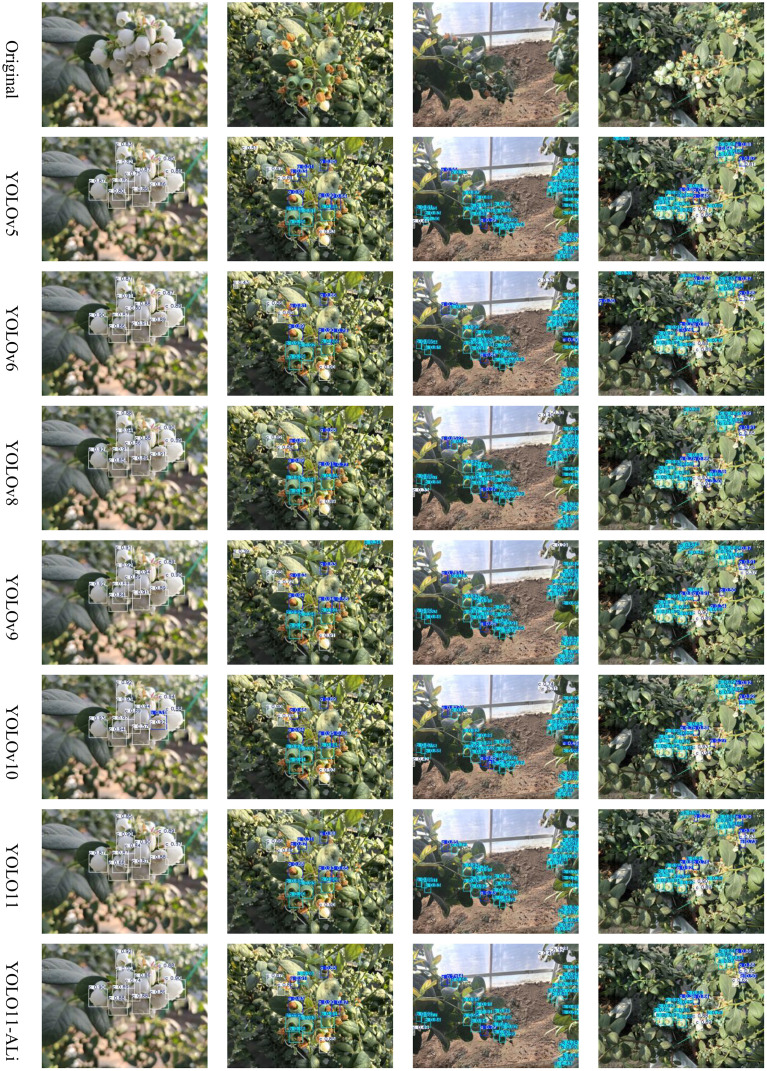
Detection Comparison Between YOLO and the Improved Model. Original; YOLOv5; YOLOv6; YOLOv8; YOLOv9; YOLOv10; YOLO11; YOLO11-ALi.

To intuitively illustrate the detailed performance of various models in detecting different categories of blueberries during the full bloom stage and fruit expansion stage, four groups of images covering three target categories were selected for visual comparison with the detection results of the aforementioned YOLO base model, as shown in **Figure 8**.

Specifically, in the first group of images, all models except YOLOv10 achieved accurate identification of open flowers, among which YOLO11-ALi yielded the highest confidence score. For the second group, although YOLO11-ALi failed to detect the open flower in the upper left corner, it neither misclassified this region as an open flower nor confused fruit with fallen flowers with that with retained flowers. In the third group, where the background was a greenhouse earthen wall under direct sunlight, only YOLO11-ALi successfully identified all three open flowers in the upper right corner. However, YOLO11-ALi exhibits a minor limitation in this scene: it incorrectly classifies a leaf in the bottom-right corner as a fruit, an error that does not appear in the original YOLO11 baseline model. Similarly, the fourth group of images demonstrated that YOLO11-ALi outperformed other models in terms of both the completeness and accuracy of flower detection. In the lower part of the upper-right clustered region, other baseline models suffer from missed detections of open flowers, whereas YOLO11-ALi achieves complete and accurate identification without omission.

In conclusion, compared with the other models, YOLO11-ALi not only delivers higher confidence in target detection under conditions of equivalent difficulty, but also enables more accurate and comprehensive identification when facing more challenging detection scenarios.

#### 3.6.2 Detection error and performance analysis

**Figure 9:**
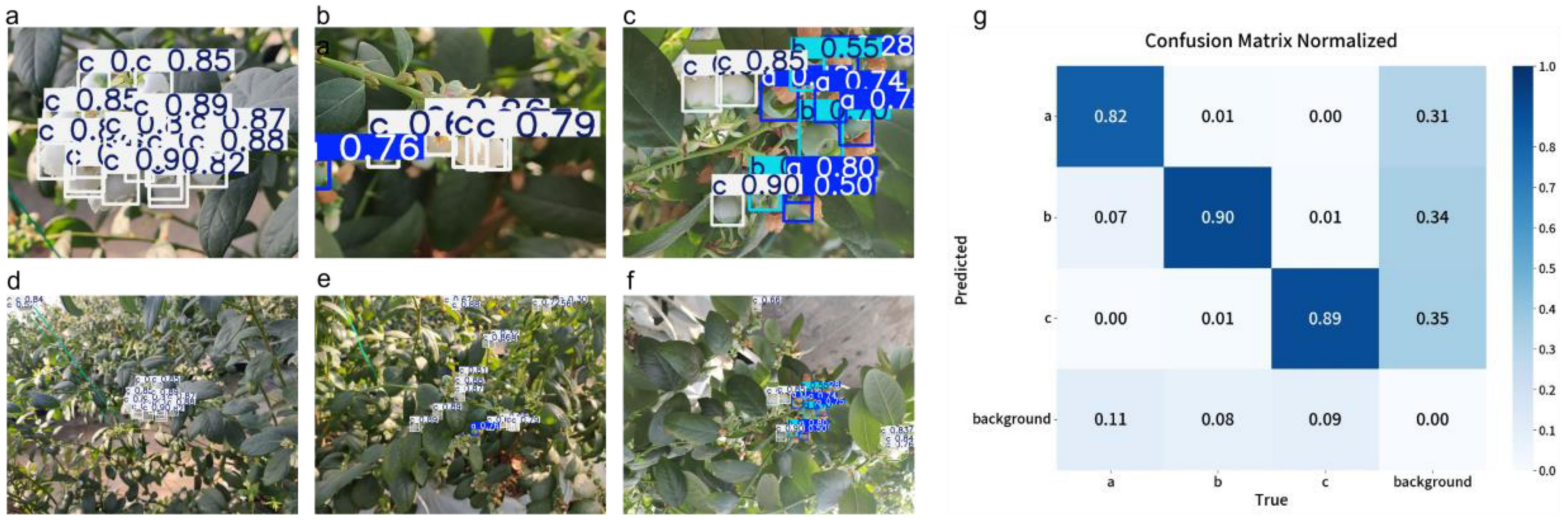
Detection results and model performance evaluation. **(a–c)** Enlarged local detection results; **(d–f)** Full-scene detection results; **(g)** Normalized confusion matrix of the model on the test set.

This study explores the model’s detection defects and classification performance in complex field environments through visual detection results and a normalized confusion matrix. The model’s performance and error sources for three blueberry growth categories are evaluated under variable illumination and diverse morphological conditions, as shown in **Figure 9**.

Images a and d show the model’s detection of dense blooming flowers under strong illumination. Intense light causes overexposure and blurred flower edges, inducing false positives and redundant detections. Images b and e present detection results in low-light conditions, where dense flower distribution also leads to detection errors. Images c and f display developing fruits with late-stage flowers nearing abscission. Although these flowers remain connected to fruits with clear spatial gaps, the model misclassifies them as fully abscised, revealing its weakness in identifying transitional floral states.

The normalized confusion matrix quantitatively validates the model’s classification performance and error patterns. The model achieves promising accuracy for all three categories. Class b refers to early-stage expanding fruits with fully abscised floral organs and exhibits the highest precision. Class c represents pre-fruiting fully bloomed flowers with slightly lower accuracy. Major errors exist between Class a and Class b. Class a denotes fruits with persistent residual floral tissues. Morphological similarities in their fruit apex structures cause mutual misclassification. Moreover, complex field backgrounds and fluctuating illumination induce minor foreground-background confusion, which is another key detection error source.

#### 3.6.3 Heatmap-based visual interpretation

**Figure 10:**
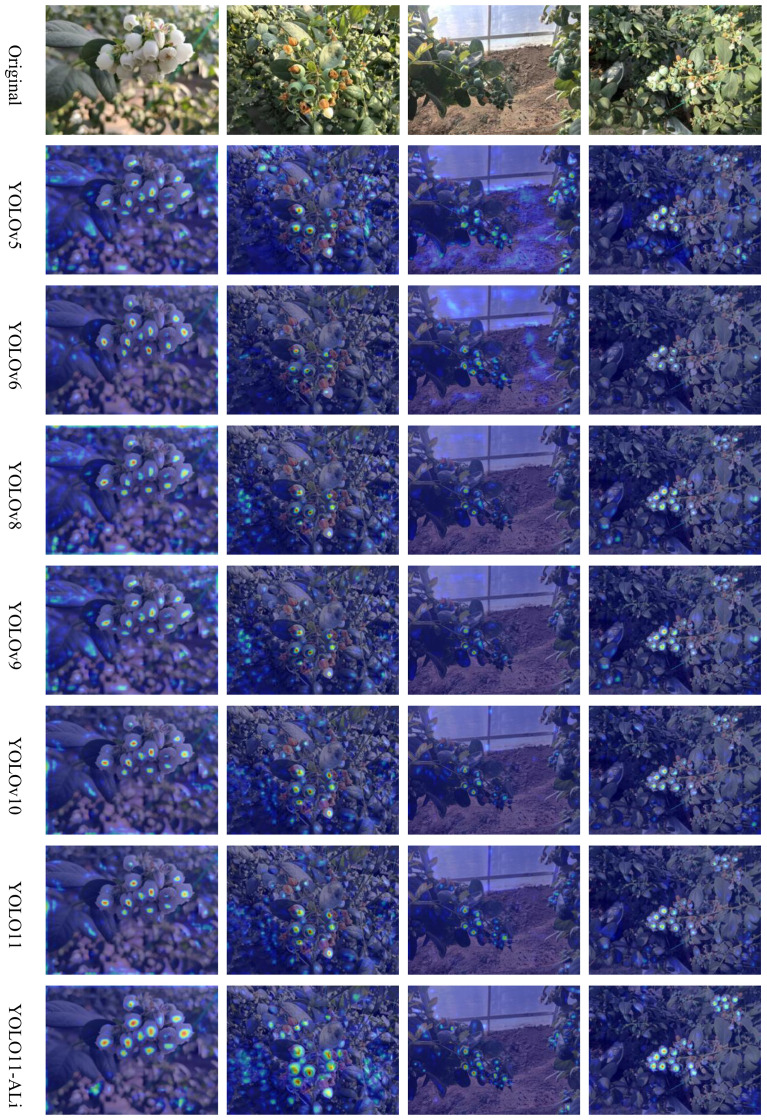
Heatmap Comparison Between YOLO and the Improved Model. Original; YOLOv5; YOLOv6; YOLOv8; YOLOv9; YOLOv10; YOLO11; YOLO11-ALi.

To further quantify and intuitively visualize the detection response characteristics of each model, the detection results of the aforementioned comparative models on the four groups of test images were visualized in the form of heatmaps, and the specific results are shown in **Figure 10**.

For the first group of images, YOLO11-ALi exhibited a significantly high-brightness response in the target regions, while no obvious activation signal was observed in the background regions, demonstrating its excellent capability to distinguish between targets and backgrounds. The heatmap results of the second group of images indicated that although YOLO11-ALi had a slightly higher degree of background activation than other comparative models, its response in the target regions was not only brighter but also covered a more complete range, highlighting a distinct advantage in target localization accuracy. In the third group of images, the differences in recognition responses of various models in the target regions were relatively small, yet YOLO11-ALi showed the lowest level of invalid background activation, reflecting its superior anti-interference performance. The heatmap features of the fourth group of images were more intuitive: YOLO11-ALi presented extremely strong high-brightness responses in the target regions, especially in the small target cluster area at the upper right corner, whereas the target activation signals of the other models were relatively weak.

In conclusion, YOLO11-ALi achieves accurate localization and efficient response for small targets in complex backgrounds, effectively balancing the sensitivity of target recognition and the specificity of background suppression.

## 4 Conclusion

This study proposes an improved detection model, YOLO11-ALi, to address the practical problem of low detection accuracy for complex targets during the critical growth stage of greenhouse substrate cultivated northern highbush blueberries, spanning from full flowering to fruit expansion. The three improved modules, namely AIFI, C2PSA_LSKA, and C3k2_iAFF, play distinct and complementary roles in blueberry target detection. The AIFI module effectively suppresses the missed detection of occluded targets by reducing the loss of small-target details. The C2PSA_LSKA module enhances the extraction of fine-grained features of blueberry targets at different growth stages through the fusion of the advantages of large and small kernel convolutions. The C3k2_iAFF module optimizes the feature representation of medium-to-high confidence targets by strengthening cross-layer feature interaction. The synergistic application of these three modules achieves a significant improvement in detection performance, verifying the rationality of the model improvement strategy. Meanwhile, the constructed dataset of northern highbush blueberries, covering three target categories during the period from full flowering to fruit expansion, effectively fills the gap in dedicated data support for this research field and provides a reliable basis for the training and validation of detection models. The YOLO11-ALi model achieves a balance between detection accuracy and real-time performance; compared with the baseline YOLO11 model, its core accuracy metrics are significantly improved, and its computational efficiency meets the deployment requirements of field portable devices.

Despite the positive outcomes achieved in this study, there are still certain limitations. The dataset was collected from a single greenhouse cultivation area, and the adaptability of the model to different blueberry varieties and regional cultivation environments needs to be further verified. Future research will expand the dataset by collecting blueberry image data from multiple regions and varieties. At the same time, lightweight optimization will be performed on the model to further reduce computational complexity and improve its deployment efficiency on edge computing devices. In addition, the integration of the detection model with agricultural Internet of Things (IoT) systems will be explored to realize the integration of blueberry growth monitoring, yield prediction, and intelligent management, thereby promoting the digital and intelligent development of the blueberry industry.

The YOLO11-ALi model can be effectively applied to the intelligent detection and monitoring of substrate cultivated northern highbush blueberries in greenhouses. By accurately identifying target categories during the stage from full flowering to fruit expansion, it provides key technical support for mitigating the negative impacts of withered flower retention on blueberry growth and quality. In addition, its high-precision recognition capability for targets in the critical growth stages of blueberries can also provide a reliable data foundation for the statistics of blueberry fruit quantity and dynamic evaluation of growth status in subsequent research, thereby assisting in the scientific estimation of blueberry yield and output value. This study offers a feasible technical approach for the refined management and decision-making of substrate-cultivated greenhouse blueberries, and further promotes the practical application value of deep learning based detection technology in protected blueberry cultivation.

## Data Availability

The raw data supporting the conclusions of this article will be made available by the authors, without undue reservation.
